# Open globe and penetrating eyelid injuries from fish hooks

**DOI:** 10.1186/s12886-019-1040-2

**Published:** 2019-01-21

**Authors:** Konstantine Purtskhvanidze, Mark Saeger, Felix Treumer, Bernhard Nölle, Johann Roider

**Affiliations:** Department of Ophthalmology, University Medical Center Schleswig-Holstein, Arnold-Heller Strasse 3, Haus 25, 24105 Kiel, Germany

**Keywords:** Ocular trauma, Open globe injury, Fish hook, Eyelid injury, Foreign body, Penetrating injury

## Abstract

**Background:**

A few case reports have described accidental eye injuries caused by fish hooks. The severity of ocular injuries is dependent on the involved ocular structures. Severe ocular injuries due to fish hooks are rare. We describe open globe and penetrating eyelid injuries from fish hooks at the Baltic Sea.

**Methods:**

Nine patients with traumatic ocular injuries caused by fish hooks were included. The following parameters were evaluated: severity of injury, best corrected visual acuity at admission and last follow-up, and surgical treatment.

**Results:**

All nine patients were male. Age ranged between 7 and 51 years with a median of 13 years. Sixty-seven percent of the patients were children. Four of the nine patients were 9 years or younger. In 5 eyes (55%) the injury was limited to the eyelid. An open globe injury was found in 4 patients (45%). The mean follow-up was 16.7 ± 32.8 months. All patients required surgical treatment. The number of operations ranged from 1 to 3, with a mean of 1.4. At admission and last follow-up, patients with eyelid injuries showed a median best corrected visual acuity (BCVA) of logMAR 0.0. Patients with open globe injuries showed a median best corrected visual acuity of logMAR 1.5 at admission, and of logMAR 0.6 at last follow-up.

**Conclusions:**

Nearly half of the patients suffered severe penetrating injuries. Especially children misjudge the risk potential of fishing due to their lack of experience. Fishing glasses should be worn not only for UV protection, but also as injury prevention strategy.

## Background

Fishing is a popular outdoor activity for people of all ages all around the world. It is considered pleasant and harmless, safety precautions are not undertaken, but nevertheless many types of accidental eye injuries caused by fish hooks can occur. Fishing is a potential cause of ocular trauma ranging from simple to severe ocular injuries. Severe ocular injuries due to fish hooks are relatively rare. The severity of ocular injuries is dependent on the involved ocular structures. Trauma caused by fish hooks may involve all structures of the eye including the lid, cornea, sclera, anterior chamber and even the posterior vitreous [1]. These injuries can be associated with subsequent traumatic cataract, vitreous hemorrhage, choroidal hemorrhage, and even retinal detachment. In certain circumstances, they can lead to endophthalmitis with partial or complete loss of vision and loss of the eye [2]. Treatment of these injuries depends on the location of injury, the involved ocular structures, and the type of hook involved. Reports of ocular injuries from fish hooks are rare and uncommon in medical literature. Mostly case reports have been published [3–6].

We describe mechanism, severity and clinical outcome of open globe and penetrating eyelid injuries by fish hooks at the Baltic Sea.

## Methods

Nine patients with traumatic ocular injuries caused by fish hooks presenting at the Department of Ophthalmology of the University Medical Center Kiel between 2005 to 2018 were included in this retrospective study. Any full-thickness injury to the cornea, sclera, or both was considered as an open globe injury. Following the Birmingham Eye Trauma Terminology (BETT) [7] injuries which included a fish hook present in the injured cornea or sclera were classified as penetrating eye injuries. The following parameters were evaluated: severity of injury, best corrected visual acuity at admission and last follow-up, and surgical treatment. These parameters were used in order to classify the open globe injuries according to the Ocular Trauma Score (OTS) [8]. The Ocular Trauma Score (OTS) developed by Kuhn et al. in 2002 is a prognostic model, which has been proposed for predicting the visual outcome based on an initial examination [8]. Kuhn et al. analyzed over 2000 eye injuries from the United States and Hungarian Eye Injury Registries, and evaluated more than 100 variables with the goal of identifying specific predictors.

## Results

**Fig. 1:**
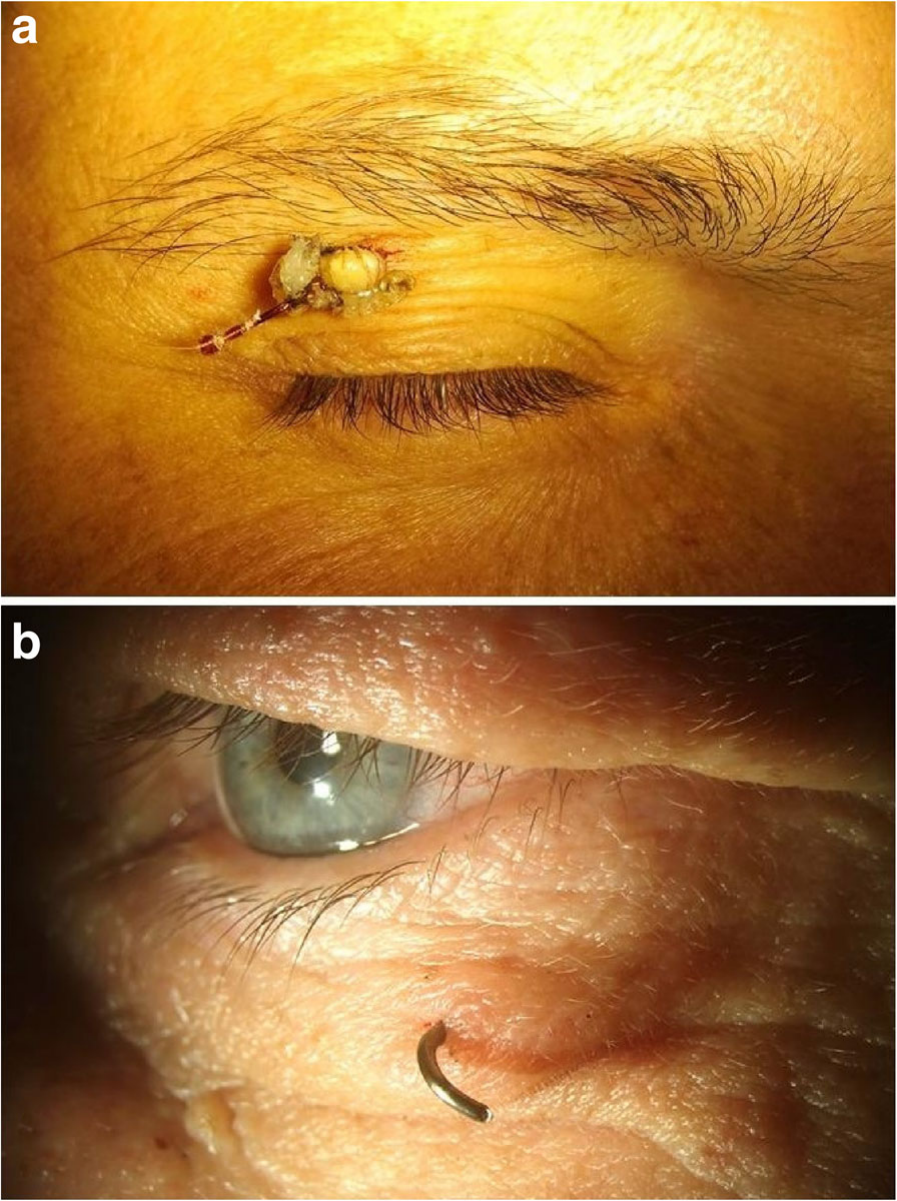
Penetrating eyelid injuries from fish hooks. **a** 37-year-old patient with a fish hook in the right upper eyelid with worm still attached. **b** 51-year-old patient presented with parts of a fish hook in his left lower eyelid. The patient cut off the fishing line and parts of the hook with a side cutter

Nine patients were treated for ocular injuries caused by fish hooks at the Department of Ophthalmology of the University Medical Center Kiel between 2005 to 2018. All 9 patients were male. Age ranged between 7 and 51 years with a median of 13 years. Sixty-seven percent of the patients were children. Four of the 9 patients were 9 years or younger. Injuries occurred in 3 right eyes (33%), and 6 left eyes (67%). All 9 accidents were related to leisure activities. Two children (22%) suffered ocular injuries while practising their fishing skills ashore. The fish hook got stuck in a tree or bush and bounced back, while pulling the line for freeing the fish hook. Eight patients were fishing (self inflicted injury), one patient suffered an injury while observing. Only one patient with an eyelid injury was wearing fishing sunglasses at the time of injury (Fig. 1b). All patients were admitted to the hospital at the day of the injury, and the median period between injury to admittance was 1 h. Five patients made no attempt to remove the fish hook and presented to the Ophthalmology Department with fish hook embedded in the eye lid, one of them with the worm still attached (Fig. 1a). One patient cut off the fishing line and parts of the hook with a side cutter (Fig. 1b). The mean follow-up was 16.7 ± 32.8 months.

### Classification of injuries

In 5 eyes (55%) the injury was limited to the eyelid. No patient suffered canalicular damage. Four patients showed an injury of the upper eyelid, 1 of the lower eyelid. An open globe injury was found in four patients (45%). Of these four patients, in one case (25%) the injury was limited to the cornea. One patient (25%) suffered an injury restricted to the sclera. In two patients (50%) the lens, iris and the posterior part of the eye was also affected.

Following BETT classification, of all nine patients, four patients displayed a penetrating injury (45%).

Two patients were assigned as an OTS 2, one as an OTS 3, and one as an OTS 4. In one patient with an OTS of 3 endopthalmitis already ocurred at admission. Table 1 presents an overview of the injured intraocular structures.

**Table 1:** Injured intraocular structures and development of visual acuity of 9 patients, injured by fish hooks from 2005 to 2018. (VB = Vitreous body, RE = Retina, CH = Choroid, LP = Light Perception)

		Visual acuity (admission)		Visual acutity (last follow-up)		Eyelid injury	Anterior segment injury				Posterior segment injury		
Age	OTS												
		logMAR	Snellen	logMAR	Snellen		Cornea	Sclera	Iris	Lens	VB	RE	CH
< 18		0.0	20/20	0.0	20/20	X							
< 18		0.0	20/20	0.0	20/20	X							
< 18		0.0	20/20	0.0	20/20	X							
≥18		0.0	20/20	0.0	20/20	X							
≥18		0.0	20/20	0.0	20/20	X							
≥18	4	1.0	2/20	0.2	12/20		X						
< 18	3	0.7	4/20	0.7	4/20			X			X		
< 18	2	2.1	LP	0.6	5/20			X	X	X	X	X	X
< 18	2	2.1	LP	1.0	2/20			X	X	X	X	X	X

### Surgical treatment

**Fig. 2:**
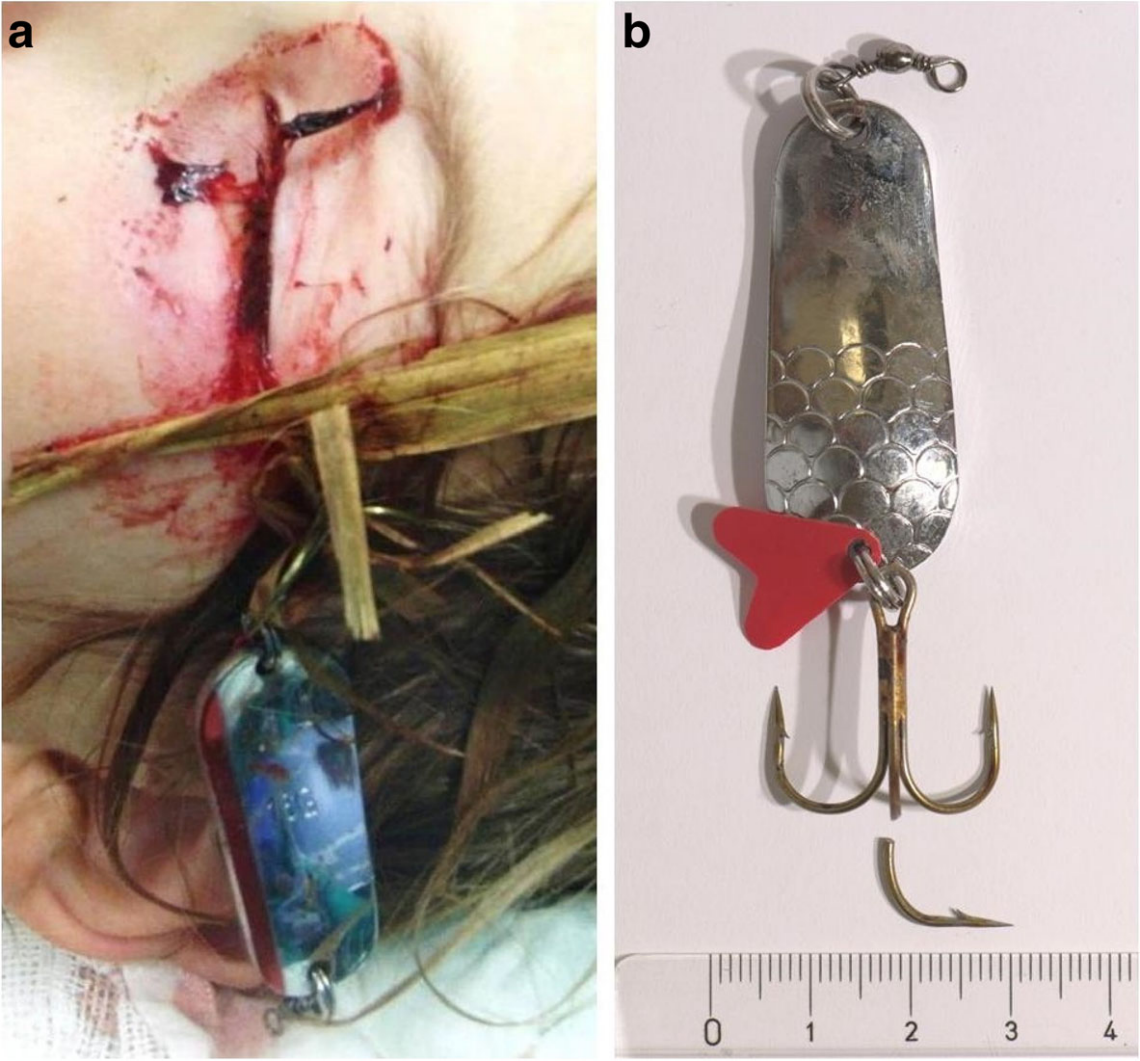
**a** Left eye of a 8-year-old patient who suffered an open globe injury while practising his fishing skills on land. The fish hook got stuck in a bush and bounced back. Parts of the plant are still attached. Visual acuity was logMAR 2.1 (LP) at admission. **b** Photograph of the fish hook (sinker’s weight 16 g) after surgical removal. (LP = Light Perception)

**Fig. 3:**
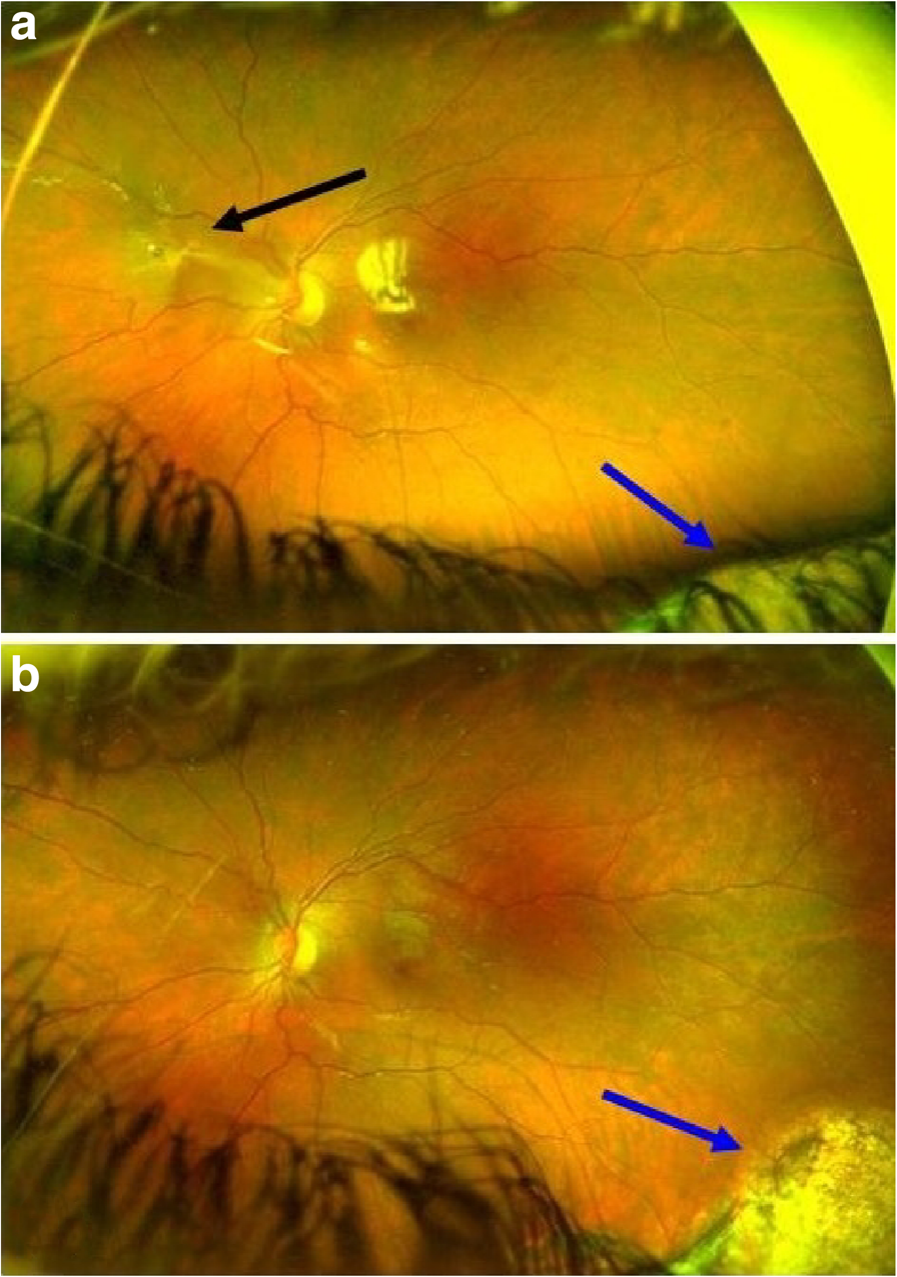
**a** Same patient as in Fig. 2. Fundus photography 3 months after injury. The black arrow indicates the PVR located nasal of the optic disc. The blue arrow indicates a chorioretinal scar. **b** 2 months after silicone oil removal and membrane peeling for PVR. No PVR detectable. The blue arrow indicates a chorioretinal scar. (PVR = proliferative vitreoretinopathy)

All patients required surgical treatment. Two patients with penetrating injuries presented without a fish hook embedded in the eye. In 5 cases, after a careful examination, the hook was removed from the eyelid at the same day under local anaesthesia. All 5 patients were treated with topical antibiotics for 1 week. In these patients, no complications were postoperatively observed. Of the 4 patients with open globe injuries, one patient only required surgery in the anterior part of the eye. This patient underwent corneal sutures with Ethilon 10–0. Another patient with a 13 mm scleral wound underwent a fish hook removal and scleral sutures (Fig. 2). Due to traumatic cataract and retinal detachment 5 days after injury, he additionally received a lentectomy, pars plana vitrectomy with silicone oil tamponade, retinal laserpexy and cryopexy. After 3 months the patient underwent pars plana vitrectomy with silicone oil removal and membrane peeling for proliferative vitreoretinopathy (Fig. 3). This patient is aphacic and wears a rigid contact lens, but a secondary lens implantation will be performed.

Two patients required a primary pars plana vitrectomy. One of these two patients with a penetrating 4 mm scleral injury 2 mm posterior to the limbus and endophthalmitis underwent a pars plana vitrectomy with antibiotic rinse and scleral sutures. The other patient had a devasting penetrating injury involving the cornea, iris and lens, an initial retinal detachment with giant retinal tear and a folded retina, and choroidal hemorrhage. The sclera was ruptured over 12 mm. This patient received corneal sutures, phacoemulsification without a primary intraocular lens implantation, a vitrectomy with silicone oil tamponade, retinal laserpexy and cryopexy. This patient also requiered a second surgery. The patient underwent pars plana vitrectomy with silicone oil exchange, membrane peeling, and retinal laserpexy. The time frame between first and second surgery was 5 days. After 6 months the patient underwent pars plana vitrectomy with silicone oil exchange, membrane peeling for proliferative vitreoretinopathy, and retinal laserpexy.

Overall, the number of operations ranged from 1 to 3, with a mean of 1.4. All patients with penetrating ocular injuries were treated with intravenously applied antibiotics for 7 days and topical antibiotic and steroid therapy for several weeks. In all patients tetanus toxoid was administered if the history of the last booster was greater than 10 years.

**Fig. 4:**
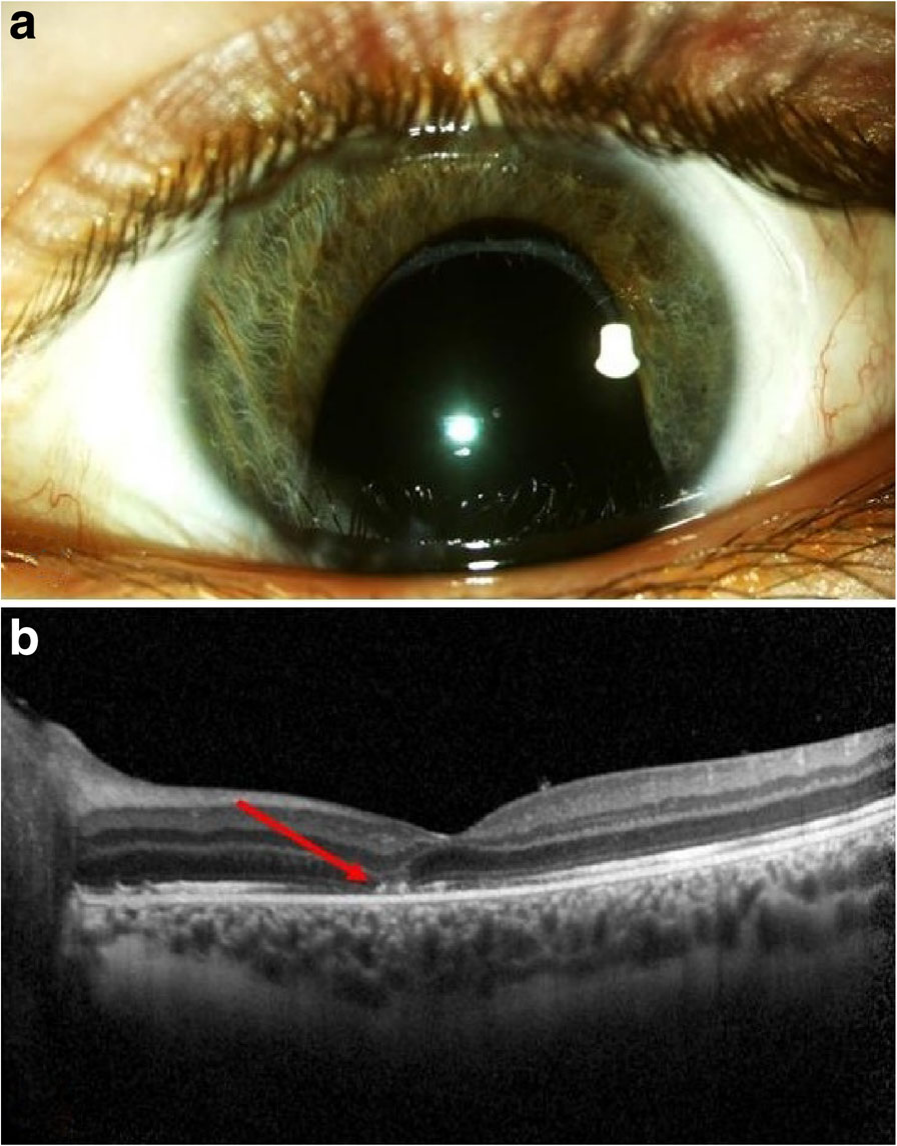
**a** Same patient as in Fig. 2. Examination 10 months after injury. Slit lamp photography: inferior iris defect and aphakia. **b** horizontal optical coherence tomography scan. The arrow indicates a photoreceptor atrophy in the fovea. At last follow-up visual acuity was logMAR of 0.6 (5/20)

In 3 patients post-traumatic complications were observed. One of these developed an optic disc atrophy. Another patient demonstrated photoreceptor atrophy in the fovea due to Berlin’s edema (Fig. 4). One patient developed a severe proliferative vitreoretinopathy with massive anterior proliferation and ocular hypotony despite silicone oil tamponade.

### Visual acuity

At admission and last follow-up (16.7 ± 32.8 months), patients with eyelid injuries showed a median best corrected visual acuity (BCVA) of logMAR 0.0. Patients with open globe injuries showed a median best corrected visual acuity of logMAR 1.5 at admission, and of logMAR 0.6 (range 1.0–0.2) at last follow-up. The development of final visual acuities basically followed the prediction made by the OTS. The individual development of BCVA is given in Table 1.

## Discussion

Fishing is a potential cause of ocular trauma. Alfaro et al. analyzed the United States Eye Injury Register (USEIR) in the period from 1998 to 2004. He reported that fishing-related eye injuries represented 19.54% of all sports-related eye injuries. Sports-related open-globe injuries occured in 44.06% [1]. The severity of the ocular trauma due to fish hooks depends on various factors, e.g. type of fish hook (barbed or barbless), velocity of the hook, direction and orientation from which it is thrown, position of the eye and eyeblink reflex to prevent the injury [9]. All ocular structures can be involved. The damage may vary from a superficial eyelid injury [3, 4] to a severe, penetrating injury [5, 6, 10]. In patients with fish hook injuries the anterior segment structures are most commonly damaged [11–13]. Severe ocular injuries due to fish hooks are rare.

In our study, nearly half of the patients suffered severe penetrating injuries. Particularly barbed fish hooks can cause severe penetrating injuries. Due to the lack of experience, especially children misjudge the potential dangers of a fish hook. That is why the majority of the fish hook injuries presenting at our department occured in children. Four of the 9 patients were 9 years or younger. Two suffered ocular injuries while practising their fishing skills. Only one patient was wearing fishing sunglasses at the time of injury and he only suffered an eyelid injury. Even though there are no particular safety precautions or warnings, it is important to keep in mind some possible complications. Eye protection is mandatory for the person fishing as well as for observers, especially young children. Fishing glasses should be worn not only for UV protection, but also as injury prevention measure. The use of eye protection may reduce the number and severity of ocular injuries from fish hooks.

## Abbreviations

BCVA: Best-corrected visual acuity
BETT: Birmingham Eye Trauma Terminology
CH: Choroid
logMAR: logarithm of minimal angle of resolution
LP: Light perception
OTS: Ocular trauma score
PVR: Proliferative vitreoretinopathy
RE: Retina
UV: Ultraviolet
VB: Vitreous body
